# Confluent and Reticulated Papillomatosis Resembling Pityriasis Versicolor and Acanthosis Nigricans: Case Report

**DOI:** 10.2196/41245

**Published:** 2023-03-02

**Authors:** Abhinav David, Divyanshu Srivastava

**Affiliations:** 1 Department of Dermatology Subharti Medical College Swami Vivekanand Subharti University Meerut India; 2 Department of Dermatology Naraina Medical College and Research Centre Kanpur India

**Keywords:** CARP, confluent and reticulated papillomatosis, pityriasis versicolor, acanthosis nigricans, minocycline

## Abstract

Confluent and reticulated papillomatosis (CARP) is a rare disorder mostly seen in young adults. It is characterized by persistent dull-brown, centrally confluent, peripherally reticulate macules and papules, which coalesce to form patches and plaques on the upper trunk and neck. It is commonly confused with pityriasis versicolor and acanthosis nigricans (AN). We report the case of a 15-year-old male with multiple pigmented confluent and reticulated patches and plaques on the neck, trunk, and chin for 3 years, which was successfully treated with oral minocycline, resulting in complete resolution of lesions within 2 weeks. The morphology of CARP resembles that of various other dermatological conditions such as AN and pityriasis versicolor, and, as a result, it is frequently misdiagnosed and mistreated, leading to social embarrassment for the patient. Therefore, it is prudent for dermatologists to carry out comprehensive clinical and histopathological assessments to facilitate prompt diagnosis and management of this condition.

## Introduction

Confluent and reticulated papillomatosis (CARP), which is also known as Gougerot-Carteaud syndrome, is an uncommon disorder of defective epidermal keratinization characterized by hyperkeratotic papules that may coalesce into confluent and reticulated plaques. It predominantly affects adolescents [1]. The etiology is unclear, but an aberrant host reaction to commensal organisms such as *Malassezia furfur* or *Dietzia papillomatosis* has been proposed [2]. CARP may masquerade as acanthosis nigricans (AN) or tinea versicolor, with few cases even showing an association with the latter [3]. We came across a case of a 15-year-old adolescent male with multiple asymptomatic pigmented lesions on the neck, upper back, chest, and chin for 3 years, which was misdiagnosed as pityriasis versicolor and AN. Treatment with oral minocycline led to successful resolution of lesions within 14 days.

## Case History

**Figure 1:**
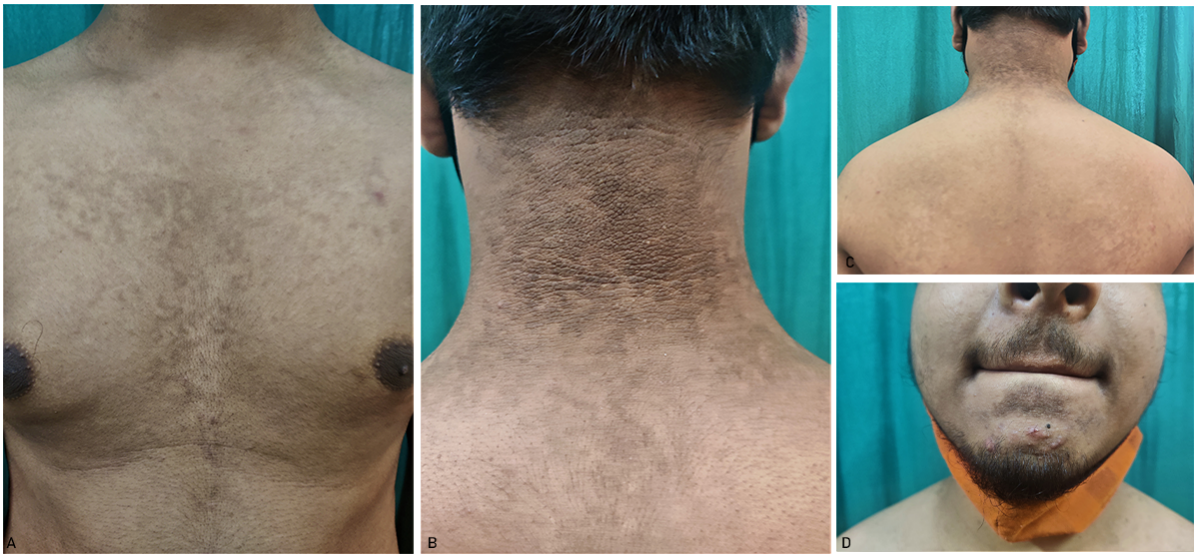
Multiple hyperpigmented macules, which are conﬂuent at the center and reticulate at the periphery, involving the (A) chest and (C) upper back. A hyperpigmented and verrucous lesion with a reticular pattern over the (B) nape of the neck and (D) chin.

**Figure 2:**
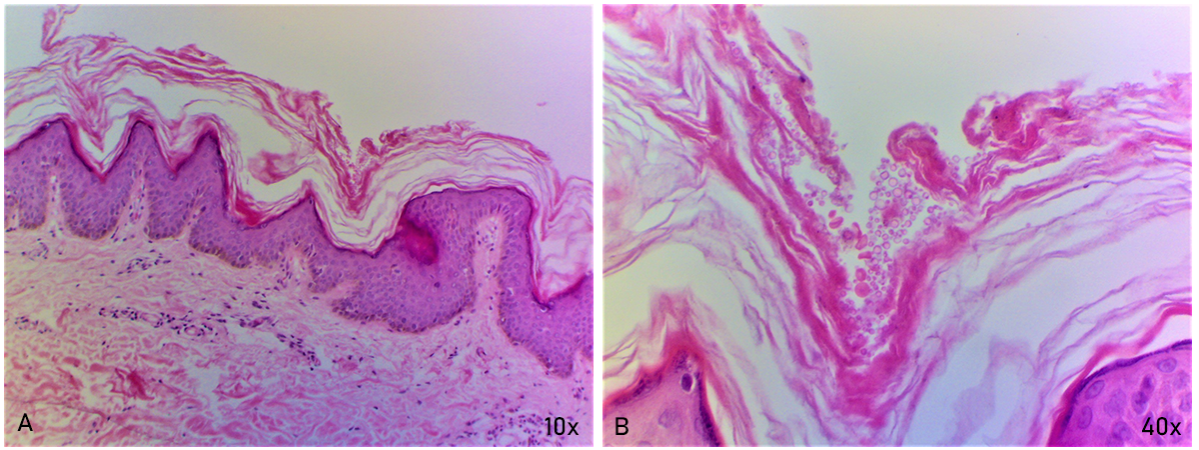
Histopathological assessment. (A) Skin section under low power (10× magnification) showing hyperkeratosis and acanthosis nigricans with low papillomatosis. The papillary dermis shows sparse perivascular lymphocytic infiltrate. (B) Few fungal spores are seen in the stratum corneum under high power (40× magnification).

A 15-year-old male presented at our outpatient department with a history of multiple, flat, hyperpigmented lesions on the neck, upper back, upper chest, and chin since the past 3 years. Lesions started on the nape of the neck, following a progressive course to involve the whole neck and upper back area. The patient complained of a rapid increase in the appearance of new lesions on the upper chest, presternal area, and chin area since the past 2 months. The lesions were asymptomatic. The patient had taken multiple unknown oral and topical medications with no effect on the size and number of lesions. There was no family history of similar lesions. His BMI was within the normal range. Dermatological examination revealed multiple hyperpigmented macules with mild scaling, which were confluent in the center and reticular toward the periphery over the neck, upper back, chest, and presternal region. Lesions over the nape of the neck and chin were hyperpigmented and verrucous (Figure 1). Differential diagnoses of CARP, AN, pityriasis versicolor, and macular amyloidosis were considered. Histopathological sections showed orthokeratotic hyperkeratosis, acanthosis, and low papillomatosis with few fungal spores in the stratum corneum. Sparse perivascular lymphocytic infiltrate was seen in the papillary and superficial dermis. Based on clinical and histopathological evaluation, a diagnosis of CARP was made (Figure 2).

**Figure 3:**
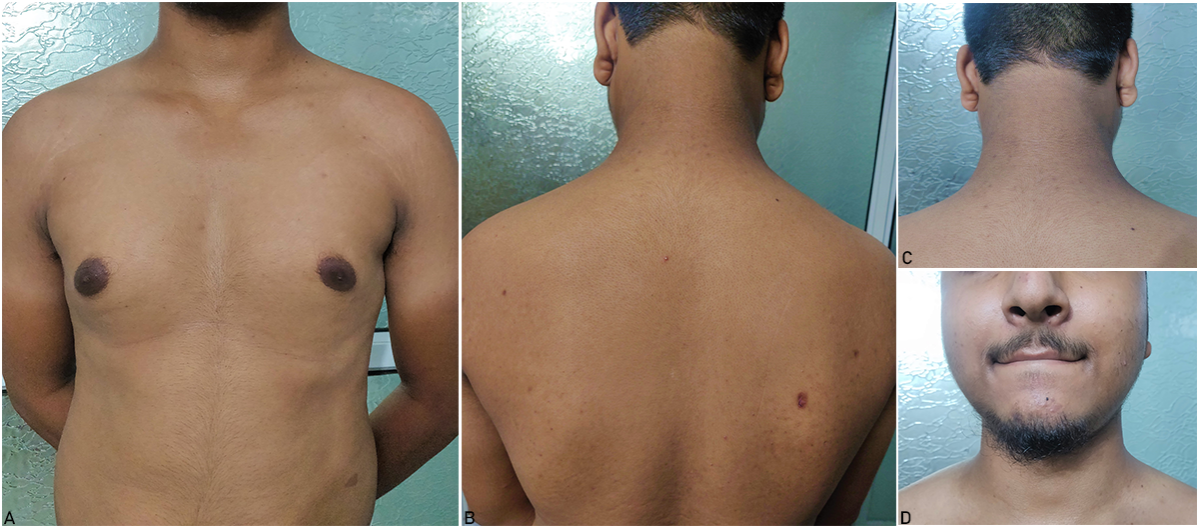
Complete resolution of all lesions post 14 days of treatment with oral minocycline on the (A) chest, (B) back, (C) nape of the neck, and (D) chin.

Oral minocycline (100 mg once daily) was initiated for the patient, and follow-up evaluation was conducted after 14 days, which revealed complete clearing of lesions from all involved sites (Figure 3).

## Discussion

### Principal Findings

CARP of Gougerot and Carteaud is a rare dermatosis seen usually in adolescents. It clinically manifests as persistent brown, scaly macules, papules, patches, or plaques. These lesions tend to be confluent at the center and become reticulated toward the periphery and are commonly located on the neck, interscapular, and intermammary regions and the axillae [4]. The etiopathogenesis of CARP is obscure till date, but data from few studies have hypothesized defective epidermal hyperkeratinization and an inherent tendency toward a hyperproliferative response to colonization by *M furfur* [5]. Recently, the presence of *Dietzia* spp in the skin has been proposed as an etiological factor in CARP. Other possible causes of CARP include endocrine abnormalities such as insulin resistance and hypothyroidism, a reaction to UV light, a variant of cutaneous amyloidosis, and genetic predisposition [1]. The histopathological characteristics of CARP include undulating basket-weave hyperkeratosis, focal acanthosis limited to the areas of elongated rete ridges, papillomatosis, and increased basal melanin pigmentation [6]. Occasionally, a mild perivascular lymphocytic infiltrate can be seen in the papillary dermis [5]. CARP is often clinically mistaken for pityriasis versicolor and usually shows no response to therapy with antifungal agents [7]. It was previously considered a clinical form of AN, but AN differs in location (axillae and neck in AN) and has a darker appearance than CARP [3,8]. Pseudoatrophoderma colli is a rare entity and is usually considered a variant of CARP. It clinically presents as atrophic and wrinkled lesions predominantly over the trunk and neck (thus named “colli”) [3].

The diagnostic criteria for CARP, as proposed by Davis et al [7] and Srinivas [9], require the following: (1) clinical presentation of scaly brown reticulated and papillomatous macules and patches; (2) the upper trunk and neck as the site; (3) negative potassium hydroxide staining of scales for spores and hyphae; (4) no response to antifungal agents; and (5) an excellent response to minocycline. 

Jo et al [10] proposed a change to the original criteria, which are as follows: (1) clinical presentation of scaly brown macules and patches, some reticulated and papillomatous; (2) the upper trunk, neck, or flexures as the site; (3) negative potassium hydroxide staining or lack of response to antifungal treatment; and (4) an excellent response to antibiotic treatment.

The main differentiating features of CARP, AN, and pseudoatrophoderma colli are summarized in Table 1 [3].

A variety of treatments for CARP are available, among which oral minocycline (50-100 mg twice daily) is the treatment of choice. Recently, azithromycin (250-500 mg thrice per week) has been used, given its better safety profile than that of minocycline. Other less effective oral treatments options for CARP include isotretinoin, acitretin, and etretinate. Various topical agents have also been used, including selenium sulfide, ketoconazole cream, tacalcitol, tazarotene, tretinoin, and calcipotriene (calcipotriol) [1].

**Table 1:** Differentiating features between confluent and reticulated papillomatosis (CARP), acanthosis nigricans (AN), and pseudoatrophoderma colli.

Features	CARP	AN	Pseudoatrophoderma colli
Age	Adolescents	Any age	15-36 years
Gender	Male predominance	Equal distribution	Female predominance
Site	Upper trunk, neck, or flexures	Neck and axillae	Trunk and neck
Clinical presentation	Scaly brown macules and patches with some appearing reticulated and papillomatous	Velvety brown plaques	Atrophic and wrinkled lesions predominantly located on the trunk and neck
Microscopic features	Undulating basket-weave hyperkeratosis, papillomatosis, focal acanthosis limited to the areas of rete ridge elongation, and increased basal melanin pigmentation	Higher degree of acanthosis and papillomatosis than CARP and pseudoatrophoderma colli, and increased melanogenesis	Loosely thickened stratum corneum, stratum spinosum of variable thickness, irregular acanthosis, hypogranulosis, and mild dermal lymphocytic infiltrate
Treatment	Excellent response to minocycline	Improvement of insulin resistance, topical retinoids, ammonium lactate, and calcipotriene	Good response to minocycline

### Conclusions

CARP is a rare dermatosis with a chronic and recurrent course, predominantly afflicting adolescents. Its etiology is controversial, but it is widely considered a disorder of keratinization. Its morphology resembles that of various other dermatological conditions such as AN and pityriasis versicolor, and, as a result, it is frequently misdiagnosed and mistreated, leading to social embarrassment for the patient. Therefore, it is prudent for dermatologists to carry out comprehensive clinical and histopathological assessments to facilitate prompt diagnosis and management of this condition. There is no standard therapy for CARP, but various therapeutic options are available such topical and systemic retinoids, oral antibiotics, topical antifungals, urea, calcipotriol, etc. Minocycline remains the first-line treatment for this condition as patients respond well to it and have minimal side effects.

## Abbreviations

AN: acanthosis nigricans
CARP: confluent and reticulated papillomatosis
